# Physicochemical quality of water and health risks associated with consumption of African lung fish (*Protopterus annectens*) from Nyabarongo and Nyabugogo rivers, Rwanda

**DOI:** 10.1186/s13104-020-4939-z

**Published:** 2020-02-10

**Authors:** Timothy Omara, Papias Nteziyaremye, Solomon Akaganyira, Dickens Waswa Opio, Lucy Nyambura Karanja, Decrah Moraa Nyangena, Betty Jematia Kiptui, Remish Ogwang, Stephen Mark Epiaka, Abigael Jepchirchir, Alfayo Maiyo

**Affiliations:** 1 Department of Chemistry and Biochemistry, School of Biological and Physical Sciences, Moi University, Uasin Gishu County, P.O. Box 3900-30100, Eldoret, Kenya; 2 Department of Quality Control and Quality Assurance, Product Development Directory, AgroWays Uganda Limited, Plot 34-60, Kyabazinga Way, P.O. Box 1924, Jinja, Uganda; 3 Africa Center of Excellence II in Phytochemicals, Textiles and Renewable Energy, Moi University, Uasin Gishu County, P.O. Box 3900-30100, Eldoret, Kenya; 4 Department of Chemistry, College of Science and Technology, University of Rwanda, P.O. Box 3900, Kigali, Rwanda; 5 Department of Mechanical Engineering, Faculty of Engineering and Architecture, American University of Beirut, P.O. Box 11-0236, Riad El-Solh, 1107 2020 Beirut, Lebanon; 6 Department of Chemistry, Faculty of Science, Kyambogo University, P.O. Box 1, Kampala, Uganda; 7 Department of Quality Control and Quality Assurance, Rene Industries Limited, P.O. Box 6034, Kampala, Uganda; 8 Department of Pharmacy, School of Medicine, College of Health Sciences, Makerere University, P.O. Box 7062, Kampala, Uganda

**Keywords:** Cancer risk, Estimated daily intake, Heavy metals, Target hazard quotient

## Abstract

**Objective:**

To determine the quality of water, heavy metal content of edible muscles of a piscivorous fish (*Protopterus annectens*) and assess the health risks associated with using water and consumption of *P. annectens* from Nyabarongo and Nyabugogo rivers of Rwanda.

**Results:**

All the water quality parameters were within World Health Organization’s acceptable limits except total nitrogen, iron, manganese and lead levels. Edible muscles of *Protopterus annectens* contained 272.8 ± 0.36, 292.2 ± 0.25, 8.8 ± 0.36, 135.2 ± 0.15, 148.0 ± 0.21 and 432. 0 ± 0.50 mg/kg of iron, manganese, copper, zinc, chromium and lead at Ruliba station and 336.0 ± 0.70, 302.6 ± 1.22, 6.4 ± 0.26, 44.7 ± 0.20, 138.2 ± 0.17 and 302.4 ± 1.50 mg/kg of iron, manganese, copper, zinc, chromium and lead at Kirinda bridge of Nyabarongo river. Health risk assessments indicated that though ingestion and dermal contact with heavy metals in water from the rivers may not cause obvious health effects, consumption of *Protopterus annectens* from Nyabarongo river may lead to deleterious health effects.

## Introduction

Environmental studies in Rwanda have reported that rivers: Mpazi, Nyabarongo, Rusine and Nyabugogo are being continuously polluted by anthropomorphic contributions [1]. Nyabugogo river pour its water into Lake Victoria and the pollution of this lake is now rated among the top ten worldwide [2]. The increasing load of contaminants has greatly deteriorated the quality of water and fish caught in Lake Victoria [3]. The presence of toxic heavy metals in water and fish poses health risks such as development of cancer, renal failure, liver damage, cardiovascular diseases and eventually death [4].

As a contribution to environmental monitoring and public health, the current study investigated the physicochemical profile of water, heavy metal content of *Protopterus annectens* and estimated the health risks associated with use of water and consumption of *P. annectens* from Nyabarongo and Nyabugogo rivers. The results were compared with reports from previous studies.

## Main text

### Method

The current study was done in Nyabarongo and Nyabugogo rivers of Rwanda. The apparatus were those previously used [5, 6]. Study approval was granted by Department of Chemistry, College of Science and Technology, University of Rwanda (Approval No. 213000076).

#### Sampling and analysis

Samples were taken from Ruliba station in Kigali (1° 58′ 37″ S and 30° 0′ 50″ E) and Kirinda bridge in Karongi district (204° 4″ S and 290° 20′ 46″ E) on Nyabarongo river. On Nyabugogo river, samples were taken from Giticyinyoni (10° 55′ 22″ S and 300° 2′ 52″ E). Water samples (*n *=1 for each site) were obtained in triplicate between April 2019 and May 2019 (10:00 to 11: 00 am, Central African Time) as described by Omara et al. [5]. Fish (6.2 to 8.1 cm; 700–903 g) were caught in triplicate from Nyabarongo river (*n *=3 for each site), identified and prepared for analysis as described before [7].

Temperature, pH and electrical conductivity of water samples were determined on-site [6]. Total, ammoniacal, nitrite and nitrate nitrogen, sulphate and phosphate contents of water samples were determined following APHA method [8]. Iron (Fe), manganese (Mn), copper (Cu), zinc (Zn), chromium (Cr), cadmium and lead (Pb) in water samples were quantified using HACH DR/2500 spectrophotometer. Fish samples were analyzed for heavy metals using a Varian AA240 atomic absorption spectrometer and results in mg/L were converted to mg/kg [7].

Quality control was performed with spiked samples analyzed once for every 10 fish samples. Recovery percentages ranged from 97.6 to 102.5%. Blanks were determined throughout the analyses and used to correct concentrations obtained. Samples were analyzed in triplicate.

#### Human health risk assessment

Average daily doses (mg/L/day) were calculated for adults (as the general population) and children (as a sensitive group) to estimate human exposure through direct ingestion (ADD_Ing_) and dermal contact (ADD_derm_) with water (Eqs. 1, 2). Estimated daily intake (EDI, mg/kg/day) for fish was calculated as described elsewhere (Eq. 3) [5, 7, 9].

1 $${\text{ADD}}_{\text{Ing}} = \frac{{C_{hm} \times W_{ir} \times E_{d} \times E_{f} }}{{W_{ab} \times T_{aet} }}$$

2 $${\text{ADD}}_{\text{derm}} = \frac{{C_{hm} \times S_{A} \times AF \times E_{d} \times E_{f} }}{{W_{ab} \times T_{aet} }}$$

3 $${\text{EDI = }}\frac{{E_{f} \times E_{d} \times F_{ir} \times C_{f} \times C_{hm} }}{{W_{ab} \times T_{aet} }}$$

where *C*_*hm*_= metal concentration in water or fish, S_A_ is exposed area = 4350 and 2800 cm^2^ for adults and children [9], W_ir_ is water ingestion rate = 21.0 and 1.8 L/day for adults and children [10], *E*_*d*_ is the exposure duration = 67 years [11], *E*_*f*_ is the exposure frequency = 365 days/year, AF is skin adherence factor = 0.7 and 0.2 mg/cm^2^/day for adults and children, *F*_*ir*_ is fresh fish ingestion = 48 g/person/day, *C*_f_ is conversion factor for fresh to dry weight for fish = 0.208, *W*_*ab*_ is average body weight = 15 kg and 60 kg for children and adults, *T*_aet_ is the average exposure time = *E*_d_ × *E*_f_ [5, 12].

Health risk index, the total risk of a non-carcinogenic element was evaluated using target hazard quotient (THQ) (Eq. 4) [5, 7, 13].

4 $${\text{THQ}} = \frac{ADD}{{R_{f} D}}\,\,{\text{or }}\,{\text{THQ}} = \frac{EDI}{{R_{f} D}}$$

where R_*f*_D is the reference dose. Since exposure to two or more toxicants results in additive and/or interactive effects, the total THQ was treated as the sum of the individual metal THQs. Carcinogenic risk, which is the product of ADD_Ing,_ ADD_derm_ or EDI and the ingestion cancer slope factor was calculated for Cr, Cd and Pb.

#### Statistical analysis

Analytical data were presented as means ± standard deviations. One way ANOVA was done followed by Tukey test (*p *<* 0.*05) using Sigma plot software (v14, Systat software Inc., USA).

### Results

Results of water and fish analyses are given in Tables 1 and 2. Toxicity indices used for risk assessments are given in Table 3, Additional files 1, 2, 3 Tables S1, S2 and S3.

**Table 1:** Hydrochemical properties of water from Nyabarongo and Nyabugogo rivers, Rwanda

Parameter	Nyabarongo river		Nyabugogo river	WHO guidelines [14]
	Ruliba station	Kirinda bridge	Giticyinyoni	
Temperature (℃)	22.6 ± 0.30	23.0 ± 0.26	22.5 ± 0.40	25.0
pH	8.15 ± 0.07	7.96 ± 0.06	8.22 ± 0.03	6.5–8.5
Conductivity (μ/Scm)	74.3 ± 3.84	20.0 ± 0.15	102.0 ± 1.0	1000
TDS (mg/L)	39.0 ± 2.0	10.0 ± 0.01	52.9 ± 0.36	2000
Total nitrogen (mg/L)	1.62 ± 0.02	1.56 ± 0.001	*12.81 ± 9.51*	3.0
Nitrite nitrogen (mg/L)	0.01 ± 0.00	0.017 ± 0.02	0.014 ± 0.003	0.1
Nitrate nitrogen (mg/L)	0.40 ± 0.01	1.39 ± 0.01	1.07 ± 0.058	10
Ammoniacal nitrogen (mg/L)	0.153 ± 0.015	0.117 ± 0.01	0.12 ± 0.006	0.5
Sulphates (mg/L)	0.58 ± 0.017	0.270 ± 0.02	0.86 ± 0.03	1000
Phosphates (mg/L)	0.42 ± 0.01	0.21 ± 0.02	0.39 ± 0.058	5.0
Fe (mg/L)	*1.61 ± 0.03*	*0.63 ± 0.02*	*1.57 ± 0.02*	0.3
Mn (mg/L)	*0.53 ± 0.002*	0.02 ± 0.002	*0.49 ± 0.03*	0.1
Cu (mg/L)	0.24 ± 0.02	BDL	0.29 ± 0.058	0.5
Zn (mg/L)	BDL	0.09 ± 0.01	0.43 ± 0.058	3.0
Cr (mg/L)	BDL	*0.06 ± 0.002*	*0.15 ± 0.00*	0.05
Cd (mg/L)	0.106 ± 0.002	BDL	BDL	0.01
Pb (mg/L)	*0.051 ± 0.01*	*0.75 ± 0.02*	*0.59 ± 0.058*	0.01

*BDL* below method detection limit

Values in italics indicate exceedance of WHO limits

**Table 2:** Heavy metal concentrations in *P. annectens* from Nyabarongo river in comparison with other global studies

Study area	Mean heavy metal concentration (mg/kg)							Year	Authors
	Fe	Mn	Cu	Zn	Cr	Cd	Pb		
Nyabarongo river, Rwanda^a^	*272.8 ± 0.36 (336.0 ± 0.70)*	*292.2 ± 0.25 (302.6 ± 1.22)*	8.8 ± 0.36 (6.4 ± 0.26)	*135.2 ± 0.15 (44.7 ± 0.20)*	*148.0 ± 0.21 (138.2 ± 0.17)*	BDL (BDL)	*432. 0 ± 0.50 (302.4 ± 1.50)*	2020	This study
Lower River Benue, Nigeria	0.36 ± 0.02	ND	ND	ND	ND	ND	0.09 ± 0.01	2018	[15]
Oguta Lake, Nigeria	ND	ND	*30.10*	ND	3.75	0.41	*18.10*	2016	[16]
Nkisa river, Nigeria	*174.66*	*11.81*	4.92	*211.33*	1.03	*0.79*	*0.98*	2014	[17]
Benin city, Nigeria.	ND	ND	ND	ND	ND	0.32^F^, *0.52*^D^	ND	2011	[18]
Anambra river, Nigeria	*60.23 ± 0.37*^b^	0.94 ± 0.06	3.01 ± 0.40	10.60 ± 0.08	0.16 ± 0.03	ND	0.01 ± 0.02	2009	[19]
	*54.60 ± 0.20*^c^	*1.00 ± 0.01*	2.86 ± 0.31	11.40 ± 0.30	0.17 ± 0.02	ND	0.01 ± 0.02		
FAO/WHO limit	30.0	1.0	30.0	40.0	10.0	0.5	0.5		[20]

Detection limits calculated with reagent blanks were 1.50, 0.20, 0.60, 2.50, 0.50, 1.14, 1.20 and 0.37 mg/kg for Fe, Mn, Cu, Zn, Cr, Cd and Pb, respectively. Values in italics are higher than their corresponding heavy metal permissible limits in fish

*ND* not determined, *F* fresh sample, *D* dry sample, *BDL* below method detection limit

^a^Results in parentheses are for *P. annectens* from Kirinda bridge; ^b, c^ results in these rows were obtained in wet and dry seasons respectively

**Table 3:** Estimated daily doses through dermal contact and ingestion of water and consumption of *P. annectens*

Indices	Group	Sampling point	Fe	Mn	Cu	Zn	Cr	Cd	Pb
ADD_Ing_ (mg/L/day)	Adults	Ruliba station	3.64E−07	1.86E−07	8.40E−08	NA	NA	3.71E−08	1.79E−08
		Kirinda bridge	2.21E−07	7.00E−09	NA	3.15E−08	2.10E−08	NA	2.63E−07
		Giticyinyoni	5.50E−07	1.72E−07	1.02E−07	1.51E−07	5.25E−08	NA	2.07E−07
	Children	Ruliba station	1.93E−07	6.36E−08	2.88E−08	NA	NA	1.27E−08	6.12E−09
		Kirinda bridge	7.56E−08	2.40E−09	NA	1.08E−08	7.2E−09	NA	9.00E−08
		Giticyinyoni	1.88E−07	5.88E−08	3.48E−08	5.16E−08	1.8E−08	NA	7.08E−08
ADD _Derm_ (mg/L/day)	Adults	Ruliba station	8.17E−05	2.69E−05	1.23E−05	NA	NA	5.38E−06	2.59E−06
		Kirinda bridge	3.20E−04	1.02E−06	NA	4.57E−06	3.05E−06	NA	3.81E−05
		Giticyinyoni	7.97E−05	2.49E−05	1.47E−05	2.18E−05	7.61E−06	NA	3.0E−05
	Children	Ruliba station	6.01E−05	1.98E−05	8.94E−06	NA	NA	3.96E−06	1.87E−06
		Kirinda bridge	2.35E−05	7.46E−07	NA	3.36E−06	2.24E−06	NA	2.80E−05
		Giticyinyoni	5.86E−05	1.83E−05	1.08E−05	1.61E−05	5.60E−05	NA	2.20E−05
EDI (mg/kg/day)	Adults	Ruliba station	4.54E−01	*4.86E−01*	1.50E−02	2.25E−01	2.46E−01	NA	*7.19E−01*
		Kirinda bridge	5.59E−01	*5.04E−01*	1.10E−02	7.4E−02	2.30E−01	NA	*5.03E−01*
	Children	Ruliba station	*1.816E0*	*1.945E0*	*5.90E−02*	9.00E−01	9.85E−01	NA	*2.88E0*
		Kirinda bridge	*2.236E0*	*2.014E0*	*4.30E−02*	2.98E−01	9.20E−01	NA	*2.01E0*
Dermal reference dose (mg/L/day) [12]			1.4E2	9.6E−01	1.2E−02	6.0E−02	6.0E−05	6.0E−05	5.25E−04
Oral reference dose (mg/kg/day) [11]			7.0E−01	3.0E−01	4.0E−02	3.0E−01	1.5E0	1.0E−03	1.4E−01

*NA* not applicable

Values in italics are higher than the reference doses of the heavy metals

### Discussion

#### Water quality

Nearly all the water quality parameters were within WHO permissible limits [14]. Temperatures were normal while the pH of samples were slightly alkaline, comparable to 7.8 reported by Usanzineza et al. [21] in Lake Muhazi. Nhapi et al. [1] reported a pH of 7.24 ± 0.18 at Rwesero, the point after which Nyabugogo river flows out of Lake Muhazi. Alkaline pH in Rwandese rivers were reported to be due to alkaline wastes from UTEXRWA industry in Kigali [22]. Overall, the pH values recorded were within WHO limits [14]. It should be noted that even within acceptable pH ranges, slightly high pH causes water to have a slippery feel whereas slightly low pH may cause water to have a bitter or metallic taste [6].

Conductivity and total dissolved solids recorded were lower than those previously reported for water from Nyabugogo marshland, Nyabugogo, Rwanzekuma and Ruganwa rivers [21]. High total dissolved solids affect the aesthetic quality of water, interferes with washing operations and can be corrosive to plumbing fixtures. Total Kjeldahl nitrogen were also low; only water from Giticyinyoni had total nitrogen higher than the maximum acceptable limit. Similarly, nitrite, nitrate and ammoniacal nitrogen levels were low. Insignificant differences in nitrite levels in water from Nyabugogo river stretch have been reported previously [1]. Presence of nitrites indicate oxidation which is influenced by environmental factors such as re-aeration, photosynthesis and presence of ammonium. Nitrate levels on the other hand were lower than those previously reported for Nyabugogo river [1]. Overall, total nitrogen in levels above acceptable limits in water may lead to low levels of dissolved oxygen affecting aquatic organisms. Thus, there is no potential contamination of water from sewage discharges and agronomic activities at the studied stations of Nyabarongo and Nyabugogo rivers.

High phosphate levels in a river indicates pollution from sewage discharges or agricultural activities [6]. In this study, low levels of phosphates and sulphates were recorded, corroborating a previous report [1] which speculated that high levels of sulphates in some sites of Nyabugogo river could be due to pollution by wastes from UTEXRWA factory.

For heavy metals, Fe, Mn and Pb were in concentrations higher than WHO limits. The high levels of Fe recorded agreed with Usanzineza et al. [21] who reported 0.756 ± 0.734 mg/L of Fe in Lake Muhazi. Nhapi et al. [1] speculated that high Fe levels in this area could be due to geological composition of its red soils and this is supported by a study [23] which reported 2896 mg/kg of Fe in soils from Nyabugogo downstream. For Pb, Nhapi et al. [1] hinted that the high levels could be due to alkaline chemicals from Nyabugogo tannery. Occurrence of Pb in the rivers could also be due to use of leaded petrol and dumping of dead lead accumulators into rivers [7]. Lead is a toxic non-essential metal that interferes with essential trace metals such as calcium and Zn. High Mn levels recorded in this study is corroborated by a preceding study which recorded 28.85 ± 23.53 mg/L of Mn in Nyabarongo stream [1]. Thus, the high Mn levels could be due to the surrounding geological formation and disturbance of soils which causes discharge of manganese-rich runoffs [1].

#### Heavy metal content of *P. annectens* muscles

Fish is migratory but heavy metal accumulation in fish is an evidence of exposure to a polluted aquatic environment. High levels of heavy metals were recorded in muscles of *P. annectens* and the chemical sequence followed was Pb > Mn > Fe > Cr > Zn > Cu > Cd at Ruliba station and Fe > Mn > Pb > Cr > Zn > Cu > Cd at Kirinda bridge (Table 2). All metal concentrations recorded except that of Cu and Cd were above FAO/WHO limits. Fish ingest heavy metals by direct uptake from water or by absorption through its organs [5, 7]. Chronic intake however depends on both external and inherent factors. Thus, the high levels of heavy metals recorded in *P. annectens* in this study could be because it is a piscivorous species [5]. The metal levels reported in *P. annectens* were higher than those previously reported except [17] which reported a concentration of 211.33 mg/kg for Zn (Table 2). Overall, differences in metal concentrations in *P. annectens* could be attributed to differences in the heavy metal concentrations of water in the studied parts of Nyabarongo river.

#### Human health risk assessment

In this study, the estimated average daily doses through ingestion and dermal contact with contaminated water ranged from 7.00 × $10^{ - 9}$ to 5.86 × $10^{ - 5}$ mg/L/day for both adults and children (Table 3). All the estimated doses were lower than corresponding reference doses for ingestion and contact with the heavy metals in water, thus no serious health risks can result from contact and consumption of water from the sampled stations of the rivers.

For consumption of *P. annectens*, EDIs ranged from 4.30 × $10^{ - 2}$ to 2.88 × $10^{0}$ mg/kg/day for both children and adults. Most EDIs exceeded individual metal reference doses, implying that there are possible health risks from consumption of *P. annectens*. For non-carcinogenic risks, THQs were all below 1 for exposure through contact and ingestion of water by both children and adults (Additional file 1: Table S1). However, THQs for Mn and Pb were above 1 for adults while only Zn and Cr had THQ < 1 for children. Thus, consumption of *P. annectens* from the studied parts of Nyabarongo river may have negative health impacts as TTHQs were greater than 1 in both adults and children.

Carcinogenic risks (CR), defined by US EPA as “the incremental probability of an individual to develop cancer, over a lifetime, as a result of exposure to a potential carcinogen” were estimated for Cr, Cd and Pb using ingestion cancer slope factor [24] (Additional file 2: Table S2). The range of risks borderline by US EPA is 1 × 10^−4^ to 1 × 10^−6^ and is unacceptable if the risks are surpassing 1 × 10^−4^ [25]. Considering intake of Cr, Cd and Pb through ingestion and dermal contact with water, the total cancer risks were below the safety level (1 × 10^−4^). Thus, there is no carcinogenic risk for both adults and children through ingestion and contact with water from the studied rivers. The CR for consumption of *P. annectens* contaminated with Cr, Cd and Pb ranged from 1.84 × 10^0^ to 3.38 × 10^2^ for both groups. These values were higher than 1 × 10^−4^, suggesting that there are potential cancer risks from consumption of *P. annectens*.

## Limitations

In this study, (i) the body weights and daily intakes were not estimated for Rwandese, (ii) ingested dose was considered to be equal to the absorbed dose, (iii) probability variables used were from US EPA guidelines which may not apply to this population, (iv) CR was estimated for Cr, Cd and Pb only because there are no CSF values for the other heavy metals investigated, (v) CSF was considered as a constant for all individuals, but this is known to vary between individuals, and (vi) the health risks were only assessed using metal toxicity in the muscles, but water and fish contain other chemicals from possible exposure routes, and metabolically active organs of *P. annectens* may contain higher heavy metal concentrations. Thus, the level of health risks may be higher than estimated in this study.

## Supplementary information

**Additional file 1: Table S1.** Target hazard quotients and non-carcinogenic health risks for ingestion and dermal contact with water and consumption of *P. annectens* from Nyabarongo and Nyabugogo rivers.

**Additional file 2: Table S2.** Cancer risks through ingestion and dermal contact with water and consumption of *P. annectens* by adults and children.

**Additional file 3: Table S3.** Raw data for physicochemical properties of water and the heavy metal content of the edible muscles of *Protopterus annectens* from Nyabarongo and Nyabugogo rivers.

## Data Availability

The datasets supporting the conclusions of this study are included within the article (and its additional files).

## Abbreviations

CR: Carcinogenic/cancer risk
EDI: Estimated daily intake
FAO: Food and Agriculture Organization of the United Nations
*P. annectens*: *Protopterus annectens*
R_*f*_D: Reference dose
THQ: Target hazard quotient
TTHQ: Total target hazard quotient
US EPA: United States Environmental Protection Agency
UTEXRWA: Usine Textile Du Rwanda
WHO: World Health Organization
