# Supplementary material for: Physicochemical quality of water and health risks associated with consumption of African lung fish (Protopterus annectens) from Nyabarongo and Nyabugogo rivers, Rwanda
Source: BMC Res Notes. 2020 Feb 10;13:66. doi: 10.1186/s13104-020-4939-z (PMC7011521; doi:10.1186/s13104-020-4939-z)
Supplement: Supplementary file 1 — Additional file 1: Table S1. Target hazard quotients and non-carcinogenic health risks for ingestion and dermal contact with water and consumption of P. annectens from Nyabarongo and Nyabugogo rivers. [file 13104_2020_4939_MOESM1_ESM.docx]

**Table S1.** Target hazard quotients and non-carcinogenic health risks for ingestion and dermal contact with water and consumption of *P. annectens* from Nyabarongo and Nyabugogo rivers.

| Sample | Group | Sampling Point | Target Hazard Quotient (THQ) | | | | | | | Total THQ |
| --- | --- | --- | --- | --- | --- | --- | --- | --- | --- | --- |
|  |  |  | Iron | Manganese | Copper | Zinc | Chromium | Cadmium | Lead |  |
| Water (ADD _Ing_) | Adults | Ruliba station | 2.60E-09 | 1.94E-07 | 7.00E-06 | NA | NA | 6.18E-04 | 3.41E-05 | 6.60E-04 |
|  |  | Kirinda bridge | 1.58E-09 | 7.29E-09 | NA | 5.25E-07 | 3.50E-04 | NA | 5.01E-04 | 8.50E-04 |
|  |  | Giticyinyoni | 3.93E-09 | 1.79E-07 | 8.50E-06 | 2.52E-06 | 8.75E-04 | NA | 3.94E-04 | 1.28E-03 |
|  | Children | Ruliba station | 1.38E-09 | 6.63E-08 | 2.40E-06 | NA | NA | 2.12E-04 | 1.17E-05 | 2.30E-04 |
|  |  | Kirinda bridge | 5.40E-10 | 2.50E-09 | NA | 1.80E-07 | 1.20E-04 | NA | 1.71E-04 | 2.90E-04 |
|  |  | Giticyinyoni | 1.34E-09 | 6.13E-08 | 2.90E-06 | 8.60E-07 | 3.00E-04 | NA | 1.35E-04 | 4.40E-04 |
| Water (ADD _Derm_) | Adults | Ruliba station | 5.83E-07 | 2.80E-05 | 1.03E-05 | NA | NA | 8.97E-02 | 4.93E-03 | 9.47E-02 |
|  |  | Kirinda bridge | 2.29E-06 | 1.06E-06 | NA | 7.62E-05 | 5.08E-02 | NA | 7.26E-02 | 1.23E-01 |
|  |  | Giticyinyoni | 5.69E-07 | 2.59E-05 | 1.23E-05 | 3.63E-04 | 1.27E-01 | NA | 5.71E-02 | 1.85E-01 |
|  | Children | Ruliba station | 4.29E-07 | 2.06E-05 | 7.45E-04 | NA | NA | 6.60E-02 | 3.56E-03 | 7.03E-02 |
|  |  | Kirinda bridge | 1.69E-07 | 7.77E-07 | NA | 5.60E-05 | 3.73E-02 | NA | 5.33E-02 | 9.07E-02 |
|  |  | Giticyinyoni | 4.19E-07 | 1.91E-05 | 9.00E-04 | 2.68E-04 | 9.30E-01 | NA | 4.19E-02 | *9.73E-01* |
| Fish (EDI*)* | Adults | Ruliba station | 6.49E-01 | **1.62E0** | 3.75E-01 | 7.50E-01 | 1.64E-01 | NA | **5.14E0** | **8.53E0** |
|  |  | Kirinda bridge | 7.99E-01 | **1.68E0** | 2.75E-01 | 2.47E-01 | 1.53E-01 | NA | **3.59E0** | **6.75E0** |
|  | Children | Ruliba station | **2.66E0** | **6.48E0** | **1.48E0** | **3.00E0** | 6.57E-01 | NA | **20.54E0** | **34.81E0** |
|  |  | Kirinda bridge | **3.19E0** | **6.71E0** | **1.08E0** | 9.93E-01 | 6.13E-01 | NA | **14.38E0** | **25.89E0** |

NA*-*Not Applicable*.* Values in bold are greater than 1.
