# Supplementary material for: Physicochemical quality of water and health risks associated with consumption of African lung fish (Protopterus annectens) from Nyabarongo and Nyabugogo rivers, Rwanda
Source: BMC Res Notes. 2020 Feb 10;13:66. doi: 10.1186/s13104-020-4939-z (PMC7011521; doi:10.1186/s13104-020-4939-z)
Supplement: Supplementary file 2 — Additional file 2: Table S2. Cancer risks through ingestion and dermal contact with water and consumption of P. annectens by adults and children. [file 13104_2020_4939_MOESM2_ESM.docx]

**Table S2.** Cancer risks through ingestion and dermal with water and consumption of *P. annectens* by adults and children

| Sample | Consumer | Sampling station | Cancer risk | | | Total cancer risk |
| --- | --- | --- | --- | --- | --- | --- |
|  |  |  | Chromium | Cadmium | Lead |  |
| Water  (Ingestion) | Adults | Ruliba station | NA | 9.76E-11 | 2.10E-09 | 2.20E-09 |
|  |  | Kirinda bridge | 4.20E-11 | NA | 3.09E-08 | 3.09E-08 |
|  |  | Giticyinyoni | 1.05E-10 | NA | 2.44E-08 | 2.45E-08 |
|  | Children | Ruliba station | NA | 3.34E-11 | 7.20E-10 | 7.53E-10 |
|  |  | Kirinda bridge | 1.44E-11 | NA | 1.06E-08 | 1.06E-08 |
|  |  | Giticyinyoni | 3.60E-11 | NA | 8.33E-09 | 8.36E-09 |
| Water  (Dermal contact) | Adults | Ruliba station | NA | 1.42E-08 | 3.05E-07 | 3.20E-07 |
|  |  | Kirinda bridge | 6.10E-09 | NA | 4.48E-06 | 4.49E-06 |
|  |  | Giticyinyoni | 1.52E-08 | NA | 3.53E-06 | 3.55E-06 |
|  | Children | Ruliba station | NA | NA | 2.20E-07 | 2.20E-07 |
|  |  | Kirinda bridge | 4.48E-09 | NA | 3.29E-06 | 3.29E-06 |
|  |  | Giticyinyoni | 1.12E-07 | NA | 2.59E-06 | 2.70E-06 |
| Fish (Consumption) | Adults | Ruliba station | **4.92E-04** | NA | **8.46E-02** | **8.51E-02** |
|  |  | Kirinda bridge | **4.60E-04** | NA | **5.92E-02** | **5.96E-02** |
|  | Children | Ruliba station | **1.97E-03** | NA | **3.39E-01** | **3.41E-01** |
|  |  | Kirinda bridge | **1.84E-03** | NA | **2.36E-01** | **2.38E-01** |
| Ingestion cancer slope factor (mg/kg/day) | | | 5.0E-04 | 3.8E-04 | 8.5E-06 |  |

N/A-Not Applicable. Values in **bold** are higher than the maximum safety limit of 1.0 × 10 ^−4^.
